# Editorial: Preventative strategies to stop the spread of antibiotic resistance

**DOI:** 10.3389/frabi.2023.1283336

**Published:** 2023-10-06

**Authors:** Santi M. Mandal

**Affiliations:** Department of Biotechnology, Indian Institute of Technology Kharagpur, Kharagpur, India

**Keywords:** antibiotic resistance, preventive strategies, artificial intelligence, digital monitoring, antibiotic stewardship

Antibiotic resistance is most burden global health issue that threatens the efficacy of antibiotics and modern medical practices. The overuse and misuse of antibiotics, along with inadequate infection control measures, have led to the rapid emergence and spread of antibiotic-resistant bacteria. Traditional strategies to combat resistance involve surveillance, stewardship, and new drug development. However, the rise in resistance cases demands innovative solutions, and digital monitoring to harnessing the artificial intelligence (AI) offers unprecedented opportunities to tackle this crisis. This Research Topic aimed to explore the recent developments in this area that need to implement and follow to stop spread of antibiotic resistance (Blázquez et al., 2018; Mandal et al., 2018; Baindara et al., 2023). Several conventional and recent strategies are collected and summarized in **Figure 1**.

**Figure 1:**
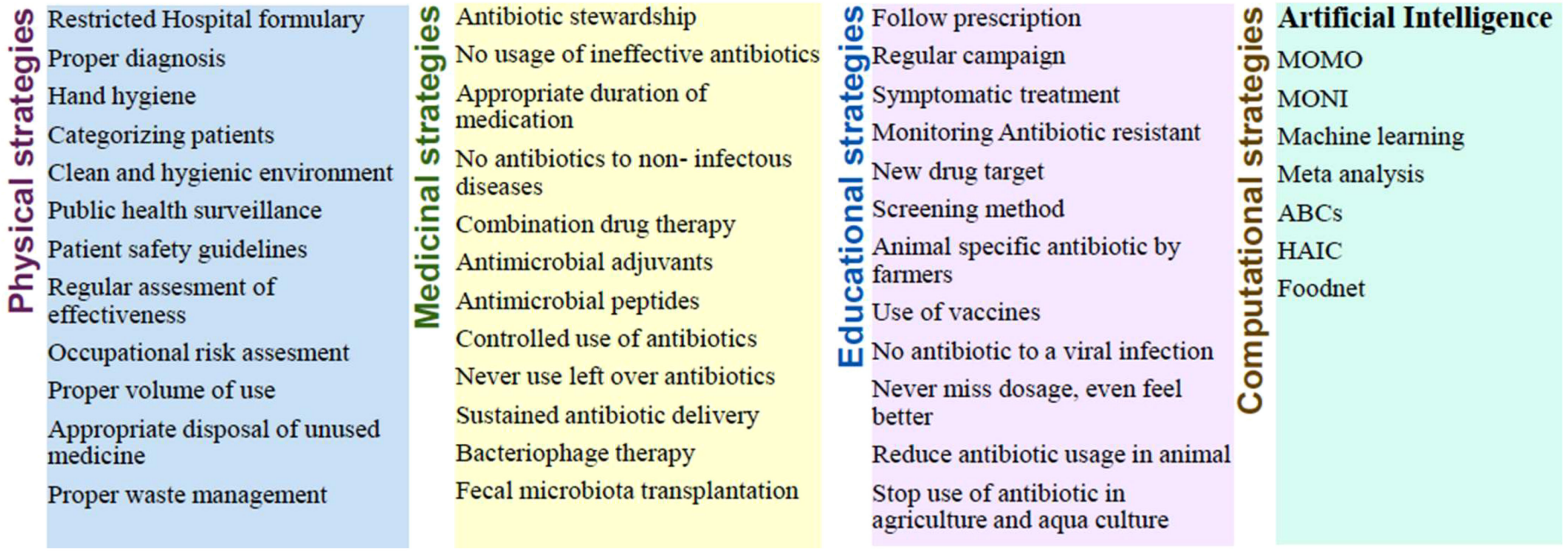
List of possible strategies to combat the spread of antibiotic resistance.

After reviewing the current literature on the effectiveness, viability, and restrictions of probiotics, including the microbiome and resistome in animal systems, Leistikow et al. noted that the use of probiotics for livestock is frequently promoted as an ideal alternative therapy that might reduce the need for the use of antibiotics in agriculture. Khalid et al. demonstrated that clinicians should use cross-sectional studies to update the antibiogram in order to assess susceptibility patterns and rationalize antibiotic empiric therapy, which will aid in lowering antibiotic resistance. Cultural sensitivity reports also enhance the rational empiric approach to administering antibiotics, which is essential for combating antimicrobial resistance. In Point Prevalence Survey (PPS) Tools, Lawry et al. described various antibiotic resistance analysis software alternatives along with their key variables for sex, gender, and pregnancy. These are important variables that affect antibiotic resistance include gender and sex. To better understand the hazards associated with pre-hospital care, these variables must be incorporated into PPS approaches. Finally, Thakral emphasizes the significance of digital technologies in healthcare settings and addresses how digitally mediated data-driven policy can be used to provide an evidence base for action in low-and middle-income countries (LMICs). In order to develop effective and sustainable antimicrobial stewardship (AMS) programs locally, the paper emphasizes the necessity of enabling networking around digital solutions, creating value in networked partnerships, launching conversations around data, and increasing awareness of the digital system.

Nevertheless, it has been shown that there is a gap in the monitoring of big data on antibiotic resistance at the local, national, and international levels. AI techniques, including machine learning and data analytics, can aid in the prediction, surveillance, diagnosis, and management of antibiotic-resistant infections (Peiffer-Smadja et al., 2020). By analyzing vast amounts of data from diverse sources, AI systems can identify patterns, predict resistance emergence, and optimize antibiotic usage. However, challenges such as data quality, ethical considerations, and integration with existing healthcare systems must be addressed. Collaborative efforts between healthcare professionals, researchers, and AI experts are crucial to maximize the impact of AI in mitigating antibiotic resistance.

## AI in antibiotic resistance surveillance

AI can enhance surveillance by analyzing vast amounts of clinical, genomic, and epidemiological data. Machine learning algorithms can identify patterns and anomalies indicative of resistance. Real-time monitoring of patient data can trigger alerts to healthcare providers when resistance is detected, allowing for swift intervention and targeted treatment.

## Prediction and early detection

AI can predict the emergence of antibiotic resistance by analyzing factors such as patient history, drug usage, and microbial genomics. Predictive models can identify high-risk populations and potential hotspots for resistance. Early detection algorithms can alert clinicians to possible resistance developments, enabling proactive measures and reducing the spread of resistant strains.

## Diagnostic support

AI-powered diagnostic tools can rapidly identify pathogens and their resistance profiles. Deep learning algorithms can analyze imaging data and molecular tests, facilitating quicker and more accurate diagnoses. This information guides appropriate treatment decisions, minimizing the unnecessary use of broad-spectrum antibiotics.

## Personalized treatment and antibiotic stewardship

AI-driven decision support systems aid clinicians in selecting the most effective antibiotic regimen for individual patients. By considering patient data and resistance patterns, these systems optimize treatment and prevent overuse of antibiotics. This supports antibiotic stewardship efforts and reduces selective pressure on bacteria.

## Data integration and interoperability

To harness the full potential of AI, integration with electronic health records and healthcare systems is essential. Standardized data formats and secure information sharing protocols are necessary to ensure seamless AI implementation across diverse healthcare settings.

## Challenges and ethical considerations

Data privacy, bias in AI algorithms, and ethical concerns regarding patient consent and autonomy must be carefully addressed. Transparency in AI decision-making is vital to build trust among healthcare professionals and patients.

## Education and awareness

AI-powered platforms can educate healthcare professionals, patients, and the general public about antibiotic resistance. These platforms can provide up-to-date information, guidelines, and recommendations for responsible antibiotic use.

## Surveillance of animal and environmental reservoirs

AI can analyze data from animals, agriculture, and the environment to monitor the spread of antibiotic resistance beyond human healthcare settings. This broader perspective can help address resistance development from its various sources.

## Regulatory and policy support

AI assist policymakers in evaluating the impact of different regulations and policies aimed at curbing antibiotic resistance. It can simulate scenarios and predict the potential outcomes of different interventions.

## Future directions

Collaboration between AI researchers, healthcare providers, epidemiologists, and policymakers is crucial to drive AI’s impact on antibiotic resistance mitigation. Longitudinal data collection, sharing, and open research initiatives are essential to train robust AI models and validate their efficacy.

In conclusion, this Research Topic provided multidisciplinary approach along with usage of probiotic, cross sectional study, sex and gender incorporation in PPS model, digital monitoring and artificial intelligence has the potential to revolutionize the way to prevent and manage antibiotic resistance. By harnessing the power of data analysis and machine learning, AI can enhance surveillance, prediction, diagnostics, and treatment optimization. Overcoming technical, ethical, and organizational challenges will determine the success of modern technologies in combating antibiotic resistance and secure the future of effective antibiotic use.

## Author contributions

SM: Conceptualization, Writing – original draft, Writing – review & editing, Investigation.
